# Perioperative systemic therapy and cytoreductive surgery with HIPEC versus upfront cytoreductive surgery with HIPEC alone for isolated resectable colorectal peritoneal metastases: protocol of a multicentre, open-label, parralel-group, phase II-III, randomised, superiority study (CAIRO6)

**DOI:** 10.1186/s12885-019-5545-0

**Published:** 2019-04-25

**Authors:** Koen P. Rovers, Checca Bakkers, Geert A. A. M. Simkens, Jacobus W. A. Burger, Simon W. Nienhuijs, Geert-Jan M. Creemers, Anna M. J. Thijs, Alexandra R. M. Brandt-Kerkhof, Eva V. E. Madsen, Ninos Ayez, Nadine L. de Boer, Esther van Meerten, Jurriaan B. Tuynman, Miranda Kusters, Nina R. Sluiter, Henk M. W. Verheul, Hans J. van der Vliet, Marinus J. Wiezer, Djamila Boerma, Emma C. E. Wassenaar, Maartje Los, Cornelis B. Hunting, Arend G. J. Aalbers, Niels F. M. Kok, Koert F. D. Kuhlmann, Henk Boot, Myriam Chalabi, Schelto Kruijff, Lukas B. Been, Robert J. van Ginkel, Derk Jan A. de Groot, Rudolf S. N. Fehrmann, Johannes H. W. de Wilt, Andreas J. A. Bremers, Philip R. de Reuver, Sandra A. Radema, Karin H. Herbschleb, Wilhelmina M. U. van Grevenstein, Arjen J. Witkamp, Miriam Koopman, Nadia Haj Mohammad, Eino B. van Duyn, Walter J. B. Mastboom, Leonie J. M. Mekenkamp, Joost Nederend, Max J. Lahaye, Petur Snaebjornsson, Cornelis Verhoef, Hanneke W. M. van Laarhoven, Aeilko H. Zwinderman, Jeanette M. Bouma, Onno Kranenburg, Iris van ‘t Erve, Remond J. A. Fijneman, Marcel G. W. Dijkgraaf, Patrick H. J. Hemmer, Cornelis J. A. Punt, Pieter J. Tanis, Ignace H. J. T. de Hingh, Dutch Peritoneal Oncology Group (DPOG), Dutch Colorectal Cancer Group (DCCG)

**Affiliations:** 1 Department of Surgery, Catharina Hospital, PO Box 1350, 5602 ZA Eindhoven, Netherlands; 2 Department of Medical Oncology, Catharina Hospital, PO Box 1350, 5602 Eindhoven, ZA Netherlands; 3 Department of Surgical Oncology, Erasmus Medical Centre, PO Box 2040, 3000 Rotterdam, CA Netherlands; 4 Department of Medical Oncology, Erasmus MC Cancer Institute, PO Box 2040, 3000 Rotterdam, CA Netherlands; 5 Department of Surgery, Amsterdam University Medical Centres, Location VUMC, PO Box 7057, 1007 Amsterdam, MB Netherlands; 6 Department of Medical Oncology, Amsterdam University Medical Centres, Location VUMC, PO Box 7057, 1007 Amsterdam, MB Netherlands; 7 Department of Surgery, St. Antonius Hospital, PO Box 2500, 3430 Nieuwegein, EM Netherlands; 8 Department of Medical Oncology, St. Antonius Hospital, PO Box 2500, 3430 Nieuwegein, EM Netherlands; 9 Department of Surgical Oncology, Netherlands Cancer Institute, PO Box 90203, 1006 Amsterdam, BE Netherlands; 10 Department of Gastrointestinal Oncology, Netherlands Cancer Institute, PO Box 90203, 1006 Amsterdam, BE Netherlands; 11 Department of Surgery, University Medical Centre Groningen, PO Box 30001, 9700 RB Groningen, RB Netherlands; 12 Department of Medical Oncology, University Medical Centre Groningen, PO Box 30001, 9700 Groningen, RB Netherlands; 13 Department of Surgery, Radboud University Medical Centre, PO Box 9101, 6500 Nijmegen, HB Netherlands; 14 Department of Medical Oncology, Radboud University Medical Centre, PO Box 9101, 6500 Nijmegen, HB Netherlands; 15 Department of Surgery, University Medical Centre Utrecht, PO Box 85500, 3508 Utrecht, GA Netherlands; 16 Department of Medical Oncology, University Medical Centre Utrecht, PO Box 85500, 3508 Utrecht, GA Netherlands; 17 Department of Surgery, Medisch Spectrum Twente, PO Box 50000, 7500 Enschede, KA Netherlands; 18 Department of Medical Oncology, Medisch Spectrum Twente, PO Box 50000, 7500 Enschede, KA Netherlands; 19 Department of Radiology, Catharina Hospital, PO Box 1350, 5602 Eindhoven, ZA Netherlands; 20 Department of Radiology, Netherlands Cancer Institute, PO Box 90203, 1006 Amsterdam, BE Netherlands; 21 Department of Pathology, Netherlands Cancer Institute, PO Box 90203, 1006 Amsterdam, BE Netherlands; 22 Department of Medical Oncology, Amsterdam University Medical Centres, Location AMC, PO Box 22660, 1100 Amsterdam, DD Netherlands; 23 Department of Clinical Epidemiology, Biostatistics & Bioinformatics, Amsterdam University Medical Centres, Location AMC, PO Box 22660, 1100 Amsterdam, DD Netherlands; 24 Clinical Trial Department, Netherlands Comprehensive Cancer Organisation (IKNL), PO Box 19079, 3501 Utrecht, DB Netherlands; 25 UMC Utrecht Cancer Centre, University Medical Centre Utrecht, PO Box 85500, 3508 Utrecht, GA Netherlands; 26 Department of Surgery, Amsterdam University Medical Centres, Location AMC, PO Box 22660, 1100 Amsterdam, DD Netherlands

**Keywords:** Colorectal neoplasms, Peritoneal neoplasms, Cytoreduction surgical procedures, Hyperthermia, induced, Neoadjuvant therapy, Adjuvant chemotherapy, Bevacizumab, Randomized controlled trial, Mortality, Progression-free survival

## Abstract

**Background:**

Upfront cytoreductive surgery with HIPEC (CRS-HIPEC) is the standard treatment for isolated resectable colorectal peritoneal metastases (PM) in the Netherlands. This study investigates whether addition of perioperative systemic therapy to CRS-HIPEC improves oncological outcomes.

**Methods:**

This open-label, parallel-group, phase II-III, randomised, superiority study is performed in nine Dutch tertiary referral centres. Eligible patients are adults who have a good performance status, histologically or cytologically proven resectable PM of a colorectal adenocarcinoma, no systemic colorectal metastases, no systemic therapy for colorectal cancer within six months prior to enrolment, and no previous CRS-HIPEC. Eligible patients are randomised (1:1) to perioperative systemic therapy and CRS-HIPEC (experimental arm) or upfront CRS-HIPEC alone (control arm) by using central randomisation software with minimisation stratified by a peritoneal cancer index of 0–10 or 11–20, metachronous or synchronous PM, previous systemic therapy for colorectal cancer, and HIPEC with oxaliplatin or mitomycin C. At the treating physician’s discretion, perioperative systemic therapy consists of either four 3-weekly neoadjuvant and adjuvant cycles of capecitabine with oxaliplatin (CAPOX), six 2-weekly neoadjuvant and adjuvant cycles of 5-fluorouracil/leucovorin with oxaliplatin (FOLFOX), or six 2-weekly neoadjuvant cycles of 5-fluorouracil/leucovorin with irinotecan (FOLFIRI) followed by four 3-weekly (capecitabine) or six 2-weekly (5-fluorouracil/leucovorin) adjuvant cycles of fluoropyrimidine monotherapy. Bevacizumab is added to the first three (CAPOX) or four (FOLFOX/FOLFIRI) neoadjuvant cycles. The first 80 patients are enrolled in a phase II study to explore the feasibility of accrual and the feasibility, safety, and tolerance of perioperative systemic therapy. If predefined criteria of feasibility and safety are met, the study continues as a phase III study with 3-year overall survival as primary endpoint. A total of 358 patients is needed to detect the hypothesised 15% increase in 3-year overall survival (control arm 50%; experimental arm 65%). Secondary endpoints are surgical characteristics, major postoperative morbidity, progression-free survival, disease-free survival, health-related quality of life, costs, major systemic therapy related toxicity, and objective radiological and histopathological response rates.

**Discussion:**

This is the first randomised study that prospectively compares oncological outcomes of perioperative systemic therapy and CRS-HIPEC with upfront CRS-HIPEC alone for isolated resectable colorectal PM.

**Trial registration:**

Clinicaltrials.gov/NCT02758951, NTR/NTR6301, ISRCTN/ISRCTN15977568, EudraCT/2016–001865-99.

## Background

The peritoneum is the second most common isolated metastatic site of colorectal cancer after the liver [1, 2]. Patients with isolated colorectal peritoneal metastases (PM) have a poor median survival, ranging from several months to approximately one year [2–6]. In the Netherlands, nearly 30 % of these patients undergo cytoreductive surgery with hyperthermic intraperitoneal chemotherapy (CRS-HIPEC) [6]. Median survival in this selected group approaches three years with a small chance of cure [7, 8]. The increasing acceptance of CRS-HIPEC in clinical practice is supported by a randomised study and several observational series [7, 9–11]. In the Netherlands, upfront CRS-HIPEC is the current standard treatment for isolated resectable colorectal PM [12]. The addition of neoadjuvant and adjuvant systemic therapy, together commonly referred to as perioperative systemic therapy, to CRS-HIPEC has potential benefits and drawbacks.

### Potential benefits of perioperative systemic therapy

Perioperative systemic therapy may eradicate systemic micrometastases. Colorectal PM mostly arise from advanced primary tumours with a high risk of systemic spread [1, 3, 4]. Indeed, systemic failure is common after CRS-HIPEC [13]. Moreover, lymph node positivity is associated with poor outcomes after CRS-HIPEC [14], probably due to higher systemic recurrence rates. Perioperative systemic therapy could improve outcomes by decreasing the risk of systemic failure.

Secondly, neoadjuvant systemic therapy may decrease the intraperitoneal tumour load. Objective morphological and histopathological responses to neoadjuvant systemic therapy are reported in about 50 and 30% of patients with colorectal PM, respectively [15, 16]. Patients with response could have favourable outcomes due to a lower intraoperative disease load, a higher chance of a complete cytoreduction, and less extensive surgery potentially leading to a lower postoperative morbidity [17, 18].

Thirdly, adjuvant systemic therapy may eradicate residual cancer cells after CRS-HIPEC. This could improve oncological outcomes by decreasing recurrence rates, as suggested by studies focusing on non-peritoneal colorectal metastases [19].

Lastly, response assessment to neoadjuvant systemic therapy could improve patient selection for CRS-HIPEC. Potentially harmful CRS-HIPEC may be avoided in patients with early progression who are unlikely to benefit due to an unfavourable tumour biology, whereas patients with a favourable response could achieve relevant long-term survival [20, 21].

### Potential drawbacks of perioperative systemic therapy

Systemic therapy appears to be less effective for colorectal PM compared to non-peritoneal colorectal metastases [22]. This phenomenon may be explained by relative insensitivity of PM to systemic treatment [23], probably as a result of a low intraperitoneal concentration of systemically administered drugs [24]. Thereby, preoperative disease progression and secondary unresectability could occur in a substantial number of patients who receive neoadjuvant systemic therapy [25, 26].

Secondly, perioperative administration of systemic therapy may decrease its reintroduction rate at disease recurrence, which occurs in the vast majority of patients [8]. As a result, perioperative systemic therapy probably only prolongs the progression-free interval without improving overall survival, as previously observed for resectable colorectal liver metastases [27, 28].

Thirdly, systemic therapy is associated with toxicity [29]. Some patients could become ineligible for CRS-HIPEC due to systemic therapy related toxicity. Moreover, preoperative administration of bevacizumab may increase postoperative complications after CRS-HIPEC [30]. Perioperative systemic therapy and its toxicity intensify and prolong the initial treatment period, which could interfere with qualify of life.

Lastly, perioperative systemic therapy and its toxicity could increase health care costs, especially in the era of increasing use of targeted agents [31, 32].

### Rationale for this study

For isolated resectable colorectal PM, there are no randomised studies that prospectively compare the oncological efficacy of perioperative systemic therapy and CRS-HIPEC with upfront CRS-HIPEC alone [33]. The available evidence solely consists of clinically heterogeneous, often non-consecutive observational studies with high risks of selection bias [33]. Notwithstanding the lack of evidence, perioperative systemic therapy is widely administered to patients with isolated resectable colorectal PM [33]. However, administration and timing of perioperative systemic therapy vary substantially between countries, hospitals, and guidelines [9, 33–35]. More importantly, it remains unknown whether perioperative systemic therapy has an intention-to-treat benefit in this setting [33–35]. Therefore, this study randomises patients with isolated resectable colorectal PM to receive either perioperative systemic therapy and CRS-HIPEC or upfront CRS-HIPEC alone.

### Rationale for perioperative systemic regimen

A total period of six months of perioperative systemic therapy is divided into three months of neoadjuvant systemic therapy and three months of adjuvant systemic therapy. A partially preoperative administration of systemic therapy could be beneficial, since some patients are unable to receive adjuvant systemic therapy due to postoperative morbidity [36]. Moreover, systemic therapy may be better tolerated before than after CRS-HIPEC, hence allowing increased dose-intensity. The potential advantages of a preoperative strategy have already been demonstrated in patients with other resectable gastrointestinal malignancies [37–39].

The rationale for the neoadjuvant regimen is derived from first-line studies in metastatic colorectal cancer. Doublet chemotherapy consisting of a fluoropyrimidine with either oxaliplatin or irinotecan achieves higher response rates than fluoropyrimidine monotherapy [40–43]. Combinations of 5-fluorouracil/leucovorin with oxaliplatin (FOLFOX), capecitabine with oxaliplatin (CAPOX), 5-fluorouracil/leucovorin with irinotecan (FOLFIRI), and capecitabine with irinotecan (CAPIRI) have a similar efficacy [44], but the latter has an unfavourable toxicity profile [45–47]. Although triplet chemotherapy achieves higher response rates than doublet chemotherapy, it substantially increases toxicity [48]. Doublet chemotherapy may therefore be preferable, since patients in this study have resectable disease without a need for aggressive conversion therapy. The efficacy of doublet chemotherapy is increased by the addition of epidermal growth factor (EGFR) inhibitors or bevacizumab [49, 50]. When added to doublet chemotherapy, similar response rates are observed for EGFR inhibitors and bevacizumab [51–53]. However, unexpectedly unfavourable outcomes were observed after addition of the EGFR inhibitor cetuximab to perioperative doublet chemotherapy for resectable colorectal liver metastases [54]. Therefore, bevacizumab seems to be the preferred targeted agent, as suggested by some observational and experimental studies focusing on colorectal PM [16, 55, 56]. It is not beneficial to add EGFR inhibitors to doublet chemotherapy with bevacizumab [57, 58]. Taken together, neoadjuvant systemic therapy in this study comprises bevacizumab with either CAPOX, FOLFOX, or FOLFIRI.

The rationale for the adjuvant regimen is derived from adjuvant studies in high-risk colon cancer. Fluoropyrimidine monotherapy is more effective than observation [59, 60], with a similar efficacy of capecitabine and 5-fluorouracil/leucovorin [61]. Addition of oxaliplatin to fluoropyrimidines is beneficial [62–64], while addition of irinotecan is not [65–68]. It is not beneficial to add targeted therapies to adjuvant chemotherapy [69–73]. Conclusively, adjuvant systemic therapy in this study consists of either CAPOX, FOLFOX, or fluoropyrimidine monotherapy.

### Rationale for phase II-III approach

This is the first prospective multicentre study in patients with isolated resectable colorectal PM in the Netherlands. Moreover, perioperative chemotherapy with bevacizumab has never been prospectively investigated in this particular patient population. As a result, little is known about the feasibility of conducting such a study and about the feasibility, safety, and tolerance of perioperative chemotherapy with bevacizumab in this setting. These issues are carefully assessed by incorporation of a randomised phase II (pilot) study, as previously successfully performed in the FOxTROT study on preoperative chemotherapy for locally advanced resectable colon cancer [74].

## Methods

This protocol summary follows the Standard Protocol Items: Recommendations for Interventional Trials (SPIRIT) Statement [75].

### Study design

This is a multicentre, open-label, parallel-group, phase II-III, superiority study that randomises eligible patients in a 1:1 ratio to receive either perioperative systemic therapy and CRS-HIPEC (experimental arm) or upfront CRS-HIPEC alone (control arm).

### Objectives

Objectives of the phase II study are to explore the feasibility of accrual, the feasibility, safety, and tolerance of perioperative systemic therapy, and the radiological and histopathological response of colorectal PM to neoadjuvant systemic therapy.

The primary objective of the phase III study is to compare survival outcomes between both arms. Secondary objectives are to compare surgical characteristics, major postoperative morbidity, health-related quality of life, and costs between both arms. Secondary objectives confined to the experimental arm are to assess major systemic therapy related toxicity and the objective radiological and histopathological response of colorectal PM to neoadjuvant systemic therapy.

### Study setting

In the phase II study, accrual, perioperative systemic therapy, and CRS-HIPEC are restricted to nine study centres. These study centres include all Dutch tertiary referral centres qualified for the surgical treatment of colorectal PM, consisting of five university hospitals and four teaching hospitals.

In the subsequent phase III study, accrual and CRS-HIPEC remain restricted to the nine study centres, whereas perioperative systemic therapy can be administered in about fifteen additional satellite centres. These satellite centres are Dutch university and (non-)teaching hospitals qualified for the systemic treatment of patients with metastatic colorectal cancer. A list of study sites can be obtained at ClinicalTrials.gov (NCT02758951).

### Eligibility criteria

#### Patients

Eligible patients are adults who have a World Health Organisation (WHO) performance status of ≤1, histological or cytological proof of PM of a non-appendiceal colorectal adenocarcinoma with ≤50% of the tumour cells being signet ring cells, resectable disease determined by abdominal computed tomography (CT) and a diagnostic laparoscopy/laparotomy, no evidence of systemic colorectal metastases within three months prior to enrolment, no systemic therapy for colorectal cancer within six months prior to enrolment, no contraindications for CRS-HIPEC, no previous CRS-HIPEC, and no concurrent malignancies that interfere with the planned study treatment or the prognosis of resected colorectal PM. Enrolled patients need to be discussed in a multidisciplinary team meeting in a study centre prior to enrolment. Importantly, enrolment is allowed for patients with radiologically non-measurable disease. The diagnostic laparoscopy/laparotomy may be performed in a referring centre, provided that the peritoneal cancer index (PCI) is appropriately scored and documented before enrolment [76].

Patients are excluded in case of any comorbidity or condition that prevents safe administration of the planned perioperative systemic therapy, determined by the treating medical oncologist (e.g. inadequate bone marrow, renal, or liver functions, previous intolerance of fluoropyrimidines or both oxaliplatin and irinotecan, dehydropyrimidine dehydrogenase deficiency, recent major cardiovascular events, bleeding diathesis, pregnant or lactating women).

#### Participating centres

Study centres should be qualified as tertiary referral centres for the surgical treatment of colorectal PM, with at least 20 procedures of CRS-HIPEC each year. Satellite centres should be qualified for the systemic treatment of patients with metastatic colorectal cancer.

### Interventions and procedures

**Fig. 1:**
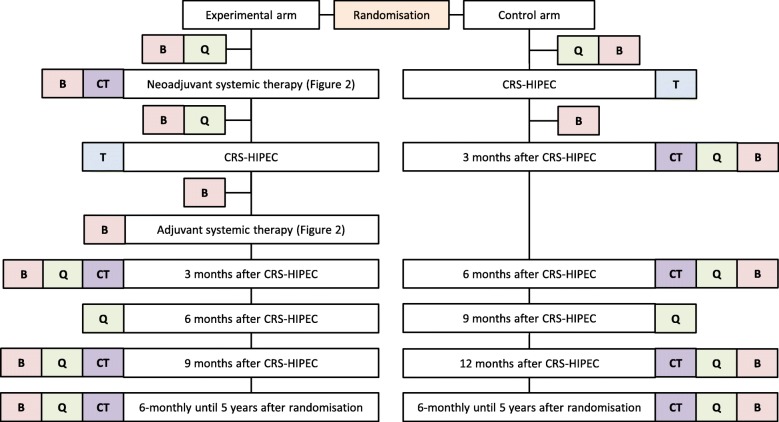
General flowchart of the CAIRO6 study. *B* blood for translational research; *CRS-HIPEC* cytoreductive surgery with hyperthermic intraperitoneal chemotherapy; *CT* thoracoabdominal computed tomography; *Q* questionnaires (EQ-5D-5L, QLQ-C30, QLQ-CR29, iMTA Productivity Cost Questionnaire, iMTA Medical Consumption Questionnaire); *T* tissue for translational research

Figure 1 shows a general flowchart of the study. Tables 1 and 2 present schedules of enrolment, interventions, and assessments of the experimental arm and the control arm, respectively.

**Table 1:** Schedule of enrolment, interventions, and assessments of the experimental arm

	Study period								
	Enrolment/allocation	Post-allocation							Close-out
	Outpatient clinics	Neoadjuvant treatment	CRS-HIPEC	Adjuvant treatment	3 months after CRS-HIPEC	6 months after CRS-HIPEC	9 months after CRS-HIPEC	Every 6 months	5 years after randomisation
Enrolment/allocation									
Eligibility screen	X								
Informed consent	X								
Allocation	X								
Interventions									
Chemotherapy		X		X					
Bevacizumab		X^a^							
CRS-HIPEC			X						
Thoracoabdominal CT		X^b^			X		X	X	X
Questionnaires	X	X^c^			X	X	X	X	X
Translational research: blood	X	X^d^	X^e^	X^d^	X		X	X	X
Translational research: tissue			X						
Assessments									
Baseline characteristics	X								
Feasibility of systemic therapy		X	X	X					
Safety/toxicity of systemic therapy		X	X	X					
Radiological response		X							
Histopathological response			X						
Surgical characteristics			X						
Postoperative morbidity			X		X				
Progression-free survival		X	X	X	X	X	X	X	X
Disease-free survival			X	X	X	X	X	X	X
Overall survival		X	X	X	X	X	X	X	X
Health-related quality of life	X	X	X		X	X	X	X	X
Costs	X	X	X		X	X	X	X	X

*CRS-HIPEC* cytoreductive surgery with hyperthermic intraperitoneal chemotherapy, *CT* computed tomography^a^Added to the first three (CAPOX) or four (FOLFOX/FOLFIRI) cycles of neoadjuvant chemotherapy

^b^After three (CAPOX with bevacizumab) or four (FOLFOX/FOLFIRI with bevacizumab) cycles

^c^After completion of neoadjuvant systemic therapy, before CRS-HIPEC

^d^Between the first and the second cycle of (neo)adjuvant systemic therapy

^e^1 day before CRS-HIPEC and 7 days after CRS-HIPEC

**Table 2:** Schedule of enrolment, interventions, and assessments of the control arm

	Study period							
	Enrolment/allocation	Post-allocation						Close-out
	Outpatient clinics	CRS-HIPEC	3 months after CRS-HIPEC	6 months after CRS-HIPEC	9 months after CRS-HIPEC	12 months after CRS-HIPEC	Every 6 months	5 years after randomisation
Enrolment/allocation								
Eligibility screen	X							
Informed consent	X							
Allocation	X							
Interventions								
CRS-HIPEC		X						
Thoracoabdominal CT			X	X		X	X	X
Questionnaires	X		X	X	X	X	X	X
Translational research: blood	X	X^a^	X	X		X	X	X
Translational research: tissue		X						
Assessments								
Baseline characteristics	X							
Surgical characteristics		X						
Postoperative morbidity		X	X					
Progression-free survival		X	X	X	X	X	X	X
Disease-free survival		X	X	X	X	X	X	X
Overall survival		X	X	X	X	X	X	X
Health-related quality of life	X	X	X	X	X	X	X	X
Costs	X	X	X	X	X	X	X	X

^a^1 day before CRS-HIPEC and 7 days after CRS-HIPEC*; CRS-HIPEC* cytoreductive surgery with hyperthermic intraperitoneal chemotherapy, *CT* computed tomography

#### Perioperative systemic therapy

**Fig. 2:**
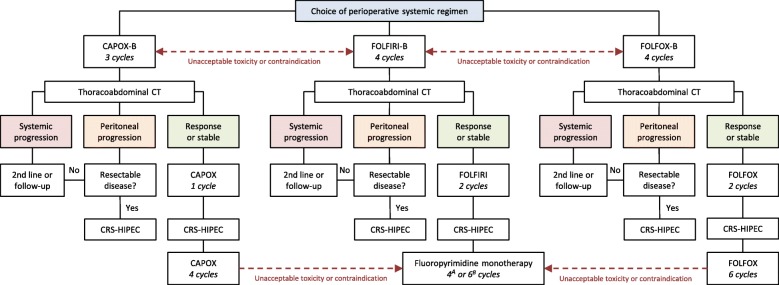
Flowchart of the perioperative systemic therapy in the experimental arm. ^A^capecitabine; ^B^5-fluorouracil, leucovorin; *CAPOX* capecitabine, oxaliplatin; *CAPOX-B* capecitabine, oxaliplatin, bevacizumab; *CRS-HIPEC* cytoreductive surgery with hyperthermic intraperitoneal chemotherapy; *CT* computed tomography; *FOLFIRI* 5-fluorouracil, leucovorin, irinotecan; *FOLFIRI-B* 5-fluorouracil, leucovorin, irinotecan, bevacizumab; *FOLFOX* 5-fluorouracil, leucovorin, oxaliplatin; *FOLFOX-B* 5-fluorouracil, leucovorin, oxaliplatin, bevacizumab

Figure 2 shows a flowchart of the perioperative systemic therapy in the experimental arm. At the discretion of the treating medical oncologist, perioperative systemic therapy consists of either:

- Four three-weekly neoadjuvant and adjuvant cycles of CAPOX (130 mg/m^2^ body-surface area [BSA] of oxaliplatin, intravenously [IV] on day 1; 1000 mg/m^2^ BSA of capecitabine, orally twice daily on days 1–14), with bevacizumab (7.5 mg/kg body weight, IV on day 1) added to the first three neoadjuvant cycles, or;
- Six two-weekly neoadjuvant and adjuvant cycles of FOLFOX (85 mg/m^2^ BSA of oxaliplatin, IV on day 1; 400 mg/m^2^ BSA of leucovorin, IV on day 1; 400/2400 mg/m^2^ BSA of bolus/continuous 5-fluorouracil, IV on day 1/1–2), with bevacizumab (5 mg/kg body weight, IV on day 1) added to the first four neoadjuvant cycles, or;
- Six two-weekly neoadjuvant cycles of FOLFIRI (180 mg/m^2^ BSA of irinotecan, IV on day 1; 400 mg/m^2^ BSA of leucovorin, IV on day 1; 400/2400 mg/m^2^ BSA of bolus/continuous 5-fluorouracil, IV on day 1/1–2) and either four three-weekly (capecitabine (1000 mg/m^2^ BSA, orally twice daily on days 1–14) or six two-weekly (400 mg/m^2^ BSA of leucovorin, IV on day 1; 400/2400 mg/m^2^ BSA of bolus/continuous 5-fluorouracil, IV on day 1/1–2) adjuvant cycles of fluoropyrimidine monotherapy, with bevacizumab (5 mg/kg body weight, IV on day 1) added to the first four neoadjuvant cycles.

Neoadjuvant systemic therapy should start within four weeks after randomisation. Adjuvant systemic therapy should start within twelve weeks after CRS-HIPEC. In case of unacceptable toxicity or contraindications to oxaliplatin or irinotecan in the neoadjuvant setting, CAPOX or FOLFOX may be switched to FOLFIRI and vice versa. In case of unacceptable toxicity or contraindications to oxaliplatin in the adjuvant setting, CAPOX of FOLFOX may be switched to fluoropyrimidine monotherapy. Dose reduction, prohibited concomitant care, permitted concomitant care, and strategies to improve adherence are not specified a priori, but left to the discretion of the treating medical oncologist. Perioperative systemic therapy can be prematurely discontinued due to radiological or clinical disease progression, unacceptable toxicity, physicians decision, or at patients request.

#### CRS-HIPEC

CRS-HIPEC is performed according to the Dutch protocol in all study centres [77]. The choice of HIPEC medication (oxaliplatin or mitomycin C) is left to the discretion of the treating physician, since neither one has a favourable safety or efficacy [78, 79]. In the control arm, CRS-HIPEC should be performed within six weeks after randomisation. In the experimental arm, CRS-HIPEC should be performed within six weeks after completion of neoadjuvant systemic therapy, and at least six weeks after the last administration of bevacizumab to minimise the risk of bevacizumab-related postoperative complications [80].

#### Follow-up

In the control arm, thoracoabdominal CT is performed three, six, and twelve months after CRS-HIPEC, and every six months thereafter until five years after randomisation. In the experimental arm, thoracoabdominal CT is performed three and nine months after CRS-HIPEC, and every six months thereafter until five years after randomisation. This follow-up schedule allows for an equal comparison of progression-free survival between both arms (Fig. 1).

#### Questionnaires

EQ-5D-5L [81, 82], QLQ-C30 [83], QLQ-CR29 [84], iMTA productivity cost questionnaire (PCQ) [85], and iMTA medical consumption questionnaire (MCQ) [86] are sent to the patients before study treatment, after completion of neoadjuvant systemic therapy (experimental arm), every three months after CRS-HIPEC until one year postoperatively, and every six months thereafter until five years after randomisation (Fig. 1).

#### Translational research – blood

An additional 20 ml blood is drawn and collected in 10 ml Cell-free DNA BCT tubes (Streck, La Vista, NE, USA) during regular blood draws before study treatment, between the first and the second cycle of neoadjuvant systemic therapy (experimental arm), one day before CRS-HIPEC, seven days after CRS-HIPEC, between the first and the second cycle of adjuvant systemic therapy (experimental arm), and every follow-up visit until disease recurrence or five years after randomisation (Fig. 1). According to the manufacturer’s instructions, collected specimens are sent to a central lab, where plasma and cell pellet are isolated and stored at − 80 °C.

#### Translational research – tissue

In all patients undergoing CRS-HIPEC, tissue specimens of colorectal PM and the primary tumour are systematically collected and stored for translational research in the study centres. Three resected colorectal PM, preferably from different regions, are collected separately. When resected, three regions of up to ±1.5 cm^3^ are excised from the primary tumour. Tumour tissue of each metastasis and each region of the primary tumour is stored as both formalin-fixed paraffin-embedded tissue and fresh frozen tissue at − 80 °C. Lastly, a piece of normal tissue is collected and stored as fresh frozen tissue at − 80 °C.

### Outcomes

Outcomes of the phase II study are to explore

- the feasibility of accrual, based on the total accrual rate, the accrual rate in each study centre, screening logs, and screening failures;
- the feasibility of perioperative systemic therapy, based on the number of patients that (1) start and complete neoadjuvant systemic therapy, with or without dose reductions, (2) are scheduled for CRS-HIPEC, (3) undergo complete CRS-HIPEC, and (4) start and complete adjuvant systemic therapy, with or without dose reductions;
- the safety of perioperative systemic therapy, based on the number of patients with (1) systemic therapy related toxicity, defined as grade ≥ 2 according to the Common Terminology Criteria for Adverse Events (CTCAE) v4.0 [87], up to one month after the last administration of systemic therapy, and (2) postoperative morbidity, defined as grade ≥ 2 according to Clavien-Dindo [88], up to three months after CRS-HIPEC;
- the tolerance of perioperative systemic therapy, based on health-related quality of life extracted from EQ-5D-5 L, QLQ-C30, and QLQ-CR29 during study treatment;
- the radiological and histopathological response of colorectal PM to neoadjuvant systemic therapy, based on central review of thoracoabdominal CT and resected specimens, respectively. Classifications are not defined a priori*.*

The primary outcome of the phase III study is 3-year overall survival, defined as the number of patients who are alive three years after randomisation. Secondary outcomes in both arms are:

- progression-free survival, defined as the time between randomisation and disease progression before CRS-HIPEC, CRS-HIPEC in case of unresectable disease, radiological proof of recurrence, or death;
- disease-free survival, defined as the time between CRS-HIPEC and radiological proof of recurrence or death;
- health-related quality of life, extracted from questionnaires (EQ-5D-5L, QLQ-C30, QLQ-CR29) at different points in time (Fig. 1, Tables 1 & 2);
- costs, derived from the Dutch costing guidelines for health care research at the time of analysis, based on case report forms, hospital information systems, and questionnaires (iMTA PCQ, iMTA MCQ, EQ-5D-5L) at different points in time (Fig. 1, Tables 1 & 2) [82];
- surgical characteristics of CRS-HIPEC (e.g. intraoperative complications, operating time, visceral and peritoneal resections, completeness of cytoreduction);
- the number of patients with major postoperative morbidity, defined as grade ≥ 3 according to Clavien-Dindo, up to three months after CRS-HIPEC.

Secondary outcomes confined to the experimental arm are:

- the number of patients with major systemic therapy related toxicity, defined as grade ≥ 3 according to the CTCAE, up to one month after the last administration of systemic therapy;
- the number of patients with an objective radiological and histopathological response of colorectal PM to neoadjuvant systemic therapy, determined by central review of thoracoabdominal CT and resected specimens, respectively. Classifications are determined after exploration of the radiological and histopathological response in the phase II study.

### Sample size

The sample size of 80 (40 in each arm) for the phase II study is chosen pragmatically as a sufficient number to explore the feasibility of accrual and the feasibility, safety, and tolerance of perioperative systemic therapy. The sample size calculation of the phase III study could only be based on a combination of low-quality observational studies [15, 16, 20, 21, 36, 57, 89–91]. A total number of 358 patients (179 in each arm) is needed to detect the hypothesised 15% increase in 3-year overall survival (control arm 50%; experimental arm 65%) with 5% drop-out, 80% power, and a two-sided log-rank test at *p* < 0.05. The primary study hypothesis may be modified when new insights or new guiding literature become available.

### Recruitment

Potential study candidates are enrolled by dedicated specialised physicians in high-volume study centres. Accrual is considered feasible when the first 80 patients are enrolled within one year after the start of accrual in the last study centre, since this ensures completion of the phase III study within a maximum of four years.

### Assignment of interventions

Eligible patients who are enrolled by physicians in study centres are centrally randomised and assigned to interventions by the coordinating investigators (KPR and CB) in a 1:1 ratio by using randomisation software (ALEA, FormsVision, Abcoude, Netherlands) with minimisation stratified by a PCI of 0–10 or 11–20, synchronous or metachronous PM, previous systemic therapy for colorectal cancer, and HIPEC with oxaliplatin or mitomycin C.

### Data collection

Questionnaires are collected by the coordinating investigators. All other baseline and outcome data are collected and entered in the central study database (TRIAS, Netherlands Comprehensive Cancer Organisation [IKNL], Utrecht, Netherlands) with electronic case report forms by independent, qualified, and trained local data managers of independent in-hospital trial departments (two study centres) or IKNL (all other centres).

### Data management

Data coding, security, and storage, including processes to promote data quality, are performed by an independent, qualified, and trained central data manager of IKNL (JMB).

### Statistical methods

Overall survival, progression-free survival, health-related quality of life, and costs are analysed in all randomised patients (intention-to-treat population). Radiological response and systemic therapy related toxicity are analysed in all patients who received at least one dose of perioperative systemic therapy (systemically treated population). Surgical characteristics, histopathological response, postoperative morbidity, and disease-free survival are analysed in all patients who receive CRS-HIPEC (operated population). Categorical variables are expressed as *n* (%). Continuous variables are expressed as mean (standard deviation) or median (range) where appropriate. All tests are two-sided and *p* < 0.05 is considered statistically significant in all analyses.

The median follow-up period is calculated by using the reverse Kaplan-Meier method. Kaplan-Meier curves of time-to-event variables are compared between both arms by using the two-sided log-rank test. Unadjusted and confounder-adjusted hazard ratios with two-sided 95% confidence intervals are estimated by using Cox proportional hazards models. Subgroup analyses are performed with stratification for relevant baseline characteristics that will be defined before the final dataset is locked. Data on patients who are event-free are censored on the date the patient is last seen. Categorical baseline characteristics and categorical outcomes are compared between both arms by using the Chi-square test or the Fisher’s exact test where appropriate. Continuous baseline characteristics and outcomes are compared between both arms by using the Mann-Whitney U test or the student’s t test where appropriate. Health-related quality of life is graphically presented across all time points and compared between both arms by using a repeated measures analysis of variance. Incremental cost-effectiveness and cost-utility ratios are calculated for the extra costs per additional patient alive and the extra costs per additional quality adjusted life year, respectively. Non-parametric bootstrapping, drawing samples of the same size as the original samples and with replacement, is applied to generate 95% confidence intervals for (differences in) costs and health outcomes. Cost-effectiveness planes are displayed and cost-effectiveness acceptability curves are drawn for willingness-to-pay values up to €100.000,-.

### Data monitoring committee

The data monitoring committee (DMC) consists of a surgeon (CV), a medical oncologist (HWML), and a statistician (AHZ), who are all independent from the sponsor and competing interests. Their role is to monitor patient safety through three interim analyses after 80 (phase II study), 160, and 240 patients complete their study treatment. Relevant data are made available to the DMC by the central data manager and the study statistician (MGAD). The study is terminated after the first interim analysis if less than 50% of the patients in the experimental arm undergo complete CRS-HIPEC or if the percentage of patients with major postoperative morbidity (Clavien-Dindo grade ≥ 3) is ≥20% higher in the experimental arm compared to the control arm. After each interim analysis, the DMC reports their advice on study continuation to the study steering committee (PHJH, CJAP, PJT, IHJTH). The study steering committee submits these reports to the ethics committee and notifies the ethics committee when (part of) the advice of the DMC is not followed. The study steering committee makes the final decision to terminate or continue the study.

### Harms

Physicians of study centres report all serious adverse events (SAEs) or suspected unexpected serious adverse reactions (SUSARs) to the coordinating investigators within 24 h. The coordinating investigators report SAEs/SUSARs through the web portal *ToetsingOnline* to the ethics committee within seven days of first knowledge for lethal or life-threatening SAEs/SUSARs, and within fifteen days for other SAEs/SUSARs. The time window for reporting SAEs/SUSARs is from randomisation to three months after CRS-HIPEC or one month after the last administration of systemic therapy. The clinical course of all SAEs/SUSARs is followed until resolution, stabilisation, or determination that study participation is not the underlying cause of the SAE/SUSAR.

### Auditing

The study is audited by independent qualified monitors of IKNL as a study with a moderate risk according to the brochure ‘Kwaliteitsborging mensgebonden onderzoek 2.0’ by the Dutch Federation of University Medical Centres. During the phase II study, each study centre is audited twice, with a focus on essential study documents, informed consent procedures, eligibility criteria, source data verification, and SAEs/SUSARs. Frequency and procedures for auditing of the phase III study are not specified and depend on auditing reports of the phase II study.

### Research ethics approval

This study is approved by the Dutch competent authority (CCMO, The Hague, Netherlands), a central ethics committee (MEC-U, Nieuwegein, Netherlands), and the institutional review boards (IRBs) of all study centres. The study will be submitted to the IRBs of the satellite centres once the accrual of the phase II study is completed.

### Protocol amendments

Important protocol modifications are communicated to all investigators, the Dutch competent authority, the central ethics committee, the IRBs of all study centres, and trial registries.

### Consent and assent

Written informed consent is obtained by physicians at the outpatient clinics of the study centres. Patients are given the possibility to give separate permission for receiving questionnaires and for participation in blood and tissue collection for translational research.

### Confidentiality

Personal information about potential and enrolled patients is collected, shared, and maintained according to the Dutch law (Wet Bescherming Persoonsgegevens) to protect confidentiality before, during, and after the study.

### Declaration of interests

The investigators declare no competing interests. Hoffman-La Roche awarded an unrestricted scientific grant for this investigator-initiated study, but has no role in the design of the study, in the collection, analysis, and interpretation of data, and in writing the manuscripts.

### Access to data

The central data manager, study statistician, coordinating investigators, and the study steering committee have access to the final datasets, without any contractual agreements that limit such access.

### Ancillary and post-study care

This study has no provisions for ancillary and post-study care. The sponsor (Catharina Hospital, Eindhoven, Netherlands) is insured to provide cover for those who suffer harm from study participation.

### Dissemination policy

Results of the phase II and phase III studies are personally communicated to participating patients. Results are communicated to healthcare professionals through publication in peer-reviewed medical journals without any publication restrictions. The manuscripts are written by the coordinating investigators, the study statistician, the study steering committee, and a professional English writer. Authorship is granted to the central data manager, the DMC, and investigators who analyse secondary outcomes (e.g. radiological or histopathological response). Authorship for physicians of study centres is granted based on the number of enrolled patients: one author for five (phase II) and twenty (phase III) patients, and an additional author for each three (phase II) and fifteen (phase III) additional patients. All other physicians and other healthcare professionals who contributed to the study are listed as collaborators. The full protocol and Dutch informed consent forms are publicly accessible [92]. Participant-level datasets and statistical codes will become available upon reasonable request.

## Discussion

This is the first randomised study that prospectively compares oncological outcomes of perioperative systemic therapy and CRS-HIPEC with upfront CRS-HIPEC alone in patients with isolated resectable colorectal PM. Results of this study will reveal whether addition of perioperative systemic therapy to CRS-HIPEC has an intention-to-treat benefit for these patients.

To the knowledge of the authors, there are no ongoing first-line or (neo)adjuvant randomised studies in metastatic colorectal cancer that could lead to modifications of the perioperative systemic therapy within the study protocol on the short term. However, there are two ongoing single arm phase II studies that investigate perioperative systemic therapy for patients with resectable colorectal PM who qualify for CRS-HIPEC. The BEV-IP study (NCT02399410) administers perioperative combination chemotherapy with bevacizumab to 45 patients with postoperative morbidity as primary outcome [93]. The CARCINOSIS study (NCT02591667) administers neoadjuvant triplet chemotherapy with bevacizumab to 35 patients with histopathological response as primary outcome. Results of these studies are actively followed to assess whether the study protocol needs to be modified. Furthermore, four studies randomise patients with colorectal peritoneal metastases after complete cytoreductive surgery: one to HIPEC with oxaliplatin or no HIPEC (PRODIGE7, NCT00769405), one to concentration-based or BSA-based HIPEC with oxaliplatin (COBOX, NCT03028155), one to HIPEC with mitomycin C or melphalan (NCT03073694), and one to HIPEC with mitomycin C or early postoperative intraperitoneal chemotherapy with leucovorin/floxuridine (ICARUS, NCT01815359). Results of these studies are closely monitored to assess whether HIPEC within the study protocol needs to be modified or omitted.

The study protocol has several potential limitations. Determination of resectable colorectal PM prior to enrolment could be difficult, since both abdominal CT and diagnostic laparoscopy tend to underestimate the PCI [94–97]. Moreover, the diagnostic laparoscopy/laparotomy prior to enrolment may also be performed in less experienced referring centres. As a result, patients with unresectable disease may be enrolled in the study. However, it is assumed that stratification by PCI equally divides these patients between both arms. Furthermore, when the diagnostic laparoscopy/laparotomy is performed in a referring centre, the PCI should be accurately scored and documented before patients can be enrolled [76]. In the future, diffusion-weighted MRI (DW-MRI) may be added to the standard preoperative work-up given its promising preliminary results in detecting resectable colorectal PM [98]. Enrolment of patients with radiologically non-measurable disease could impede response assessment to neoadjuvant systemic therapy [99]. However, since non-measurable colorectal PM are frequently observed in clinical practice, especially in patients with a low PCI or metachronous PM, the investigators decided to allow enrolment of these patients in order to create a representative study population. Enrolment is also allowed for patients who are referred to a study centre after a macroscopically complete resection of colorectal PM in a referring centre, since it is assumed that microscopic (and often macroscopic) colorectal PM are still present.

The study protocol has potential strengths. The accrual is expected to be feasible, since potential study candidates are seen by dedicated specialised physicians in all Dutch high-volume centres. Moreover, patients with isolated resectable colorectal PM do not qualify for any other multicentre randomised study in the Netherlands. The phase II-III approach allows for adequate monitoring of the feasibility and safety of perioperative systemic therapy in this setting. Extensive assessment of health-related quality of life and costs could help to standardise the treatment paradigm in the era of value-based medicine, whereas translational side studies may open new avenues for research in the era of increasing insights in the different molecular subtypes of colorectal cancer.

## Abbreviations

*BSA*: Body surface area
*CAPIRI*: Capecitabine, irinotecan
*CAPOX*: Capecitabine, oxaliplatin
*CRS*: Cytoreductive surgery
*CRS-HIPEC*: Cytoreductive surgery with hyperthermic intraperitoneal chemotherapy
*CT*: Computed tomography
*CTCAE*: Common terminology criteria for adverse events
*DMC*: Data monitoring committee
*EGFR*: Epidermal growth factor receptor
*FOLFIRI*: 5-fluorouracil, leucovorin, irinotecan
*FOLFOX*: 5-fluorouracil, leucovorin, oxaliplatin
*HIPEC*: Hyperthermic intraperitoneal chemotherapy
*IKNL*: Integraal Kankercentrum Nederland (Netherlands Comprehensive Cancer Organisation)
*IRB*: Institutional review board
*IV*: Intravenously
*MCQ*: Medical consumption questionnaire
*PCI*: Peritoneal cancer index
*PCQ*: Productivity cost questionnaire
*PM*: Peritoneal metastases
*SAE*: Serious adverse event
*SPIRIT*: Standard protocol items: recommendations for interventional trials
*SUSAR*: Suspected unexpected serious adverse reaction
*WHO*: World health organisation
